# Supplementary material for: Ultra-low noise optical injection locking amplifier with AOM-based coherent detection scheme
Source: Sci Rep. 2018 Sep 3;8:13135. doi: 10.1038/s41598-018-31381-x (PMC6120882; doi:10.1038/s41598-018-31381-x)
Supplement: Supplementary file 3 — LaTeX Supplementary File [file 41598_2018_31381_MOESM3_ESM.zip › SREP-18-11681/suppl_data/SREP-18-11681-s05.pdf]

Ultra-low noise optical injection locking amplifier with AOM-based coherent detection scheme

Zitong Feng[1,3], Yang Fei[1,*], Xi Zhang[1,3], Dijun Chen[1], Fang Wei[1], Nan Cheng[2], Yanguang Sun[1], Youzhen Gui[2], and Haiwen Cai[1,*]

[1]Shanghai Key Laboratory of All Solid-State Laser and Applied Techniques, Shanghai Institute of Optics and Fine Mechanics, Chinese Academy of Sciences, Shanghai, 201800, China
[2]Key Laboratory for Quantum Optics, Shanghai Institute of Optics and Fine Mechanics, Chinese Academy of Science, Shanghai, 201800, China
[3]University of Chinese Academy of Sciences, Beijing, 100049, China
[*]fyang@siom.ac.cn
[*]hwcai@siom.ac.cn

ABSTRACT

A novel optical injection locking amplifier with acousto-optic modulator based phase modulation and a coherent detection scheme for optical frequency transfer applications is experimentally demonstrated in this study. A commercial distributed feedback diode laser is injection-locked to the resonant frequency of the optical signal with an optical fiber path length of hundreds of kilometers. This provides approximately 59 dB gain and ensures that the input carrier frequency fractional stability can be as good as 10^{-20} at 1000 s. The amplifier was tested for the transfer of a commercial narrow-linewidth laser in a 180 km fiber link to a remote site with only a single amplification step. The transferred frequency at the remote end reached 10^{-20} at 20000 s, which is suitable for optical frequency distribution and remote comparison between optical atomic clocks.

Introduction

Immense progress in the development of frequency standards has facilitated high performance in various research fields . State-of-the-art optical frequency standards are superior to the current cesium-based atomic clocks, in which a fractional systematic uncertainty can reach as low as 10^{-18}[1,2], and the fractional instabilities in the same regime are below 10^{-17}[3,4]. They serve as frequency references for testing basic theories such as quantum electrodynamics, general relativity, and their foundations, for example, to verify the constancy of the fundamental constants and other experiments that can be performed locally[5,6]. However, studies in fundamental physics, navigation, time keeping, or geodetic applications[7,8,19] require that the clock signal be transferred to remote sites. Traditional satellite-based frequency transfer is far from meeting the requirements as its stability is only 10^{-16} over an entire day. Therefore, optical fiber links have been widely investigated as possible media for transferring today's most stable existing frequencies to remote locations[9,10,16,20–26]. In order to transmit an optical signal across a distance up to thousands of kilometers, the noise introduced by the optical fiber link should be eliminated and the power loss caused by long-distance transmission should be compensated in order to significantly improve the signal-to-noise ratio of the optical signal detected at both the local and remote end.

An erbium-doped fiber amplifier (EDFA) is the most commonly used amplifier for amplification in an optical fiber link. However, the bidirectional nature of the frequency transfer system (Doppler phase noise cancellation technology) limits the gain of the system and deteriorates the stability of transmission; this is because the point reflections and Rayleigh scattering in the fiber link may send feedback into the EDFAs, which may trigger the stimulated effect[11,12]. Fiber Brillouin amplification (FBA) is an alternative technique that can provide an amplification gain of 50 dB with approximately 30 mW pump power only. Further, since the pump laser frequency will be locked at the signal laser frequency to achieve a narrow gain bandwidth (i.e., quasi-unidirectional amplification), Rayleigh scattering and point reflections have negligible effects during transmission of the optical frequency[13,14]. Kim et al.[15] proposed an optical injection phase-locked loop (OIPLL) scheme that can provide a high net gain of approximately 44 dB with narrow gain bandwidth when an optical signal is transmitted through a 146 km fiber link. They obtained the phase noise of the optical injection locking system based on intensity modulation and direct detection, and they realized the locking effect by driving the pump current in the slave laser using an electronic feedback loop with a 750 Hz feedback bandwidth.

An amplifier with better performance is urgently needed with stability much better than 10^{-18} at 1000 s to meet the transmission of optical frequency standards[1]. In this article, we propose a novel OIPLL structure with four improvements. 1)

A different type of distributed feedback (DFB) diode laser is used as the slave laser so that the amplifier can have a higher output power of 10 dBm to 15 dBm. 2) The actuator device used to execute OIPLL feedback is an acousto-optic modulator (AOM), which can provide a feedback loop with higher bandwidth and hence improve noise suppression. 3) The error signal used in the OIPLL is obtained by phase modulating the AOM signal combined with coherent detection, which can provide higher detection sensitivity. 4) By combining the polarization-maintaining (PM) structure within the OIPLL, the system can be more robust without an additional polarization controller. As a result, we built an optical injection locking amplifier (OILA) with approximately 59 dB gain and frequency stability as good as 10^{-20} at 1000 s.

Results

Optical injection locking amplifier (OILA)

The OILA configuration is shown in Figure 1. A weak optical carrier ω_0, as shown in the black box (a), is divided into two parts (A and B) by an optical coupler. Part A is used as a reference while part B passes through an AOM with a PM fiber. The frequency thus shifts to $\omega_0 + \omega_{AOM} + \Delta\dot{\varphi}_c$, as shown in (b), where ω_{AOM} is the fixed 80 MHz frequency shift and $\Delta\dot{\varphi}_c$ is the frequency shift of the AOM generated by the control signal of the proportional-integral (PI) circuit. Light then passes through a PM three-port cyclic optical circulator and is injected into a commercial DFB diode laser ("slave" laser with optical frequency ω_s), which is shown in the black box (c). Since the optical fibers between the AOM and DFB are PM fibers, we did not include an additional polarization controller between the AOM and the optical circulator. Inside the DFB laser, if the detuning frequency $\Delta\omega_{inj} = \omega_0 + \omega_{AOM} + \Delta\dot{\varphi}_c - \omega_s$ is within the optical injection locking bandwidth, the optical frequency of this "slave" laser becomes locked at the optical frequency of the injected signal, and a frequency fluctuation of $\Delta\dot{\varphi}_L$ due to the injection locking process gets added at point C. The signal $\omega_0 + \omega_{AOM} + \Delta\dot{\varphi}_c + \Delta\dot{\varphi}_L$ at point D again gets divided into two parts. One part is an amplified output signal, and the other part is compared with the reference signal in a 50/50 optical coupler, as shown in the black frame (d). Two optical signal beats at the photodiode (PD) generate an error signal $\omega_{AOM} + \Delta\dot{\varphi}_c + \Delta\dot{\varphi}_L$; the signal is detected using single sideband coherent detection with high sensitivity. The RF signal after amplification and filtering is mixed with the 80 MHz reference signal through an analog RF phase discriminator. The output of the phase fluctuations $\Delta\dot{\varphi}_c + \Delta\dot{\varphi}_L$ (error signal) will be dealt with a PI algorithm to make $\Delta\dot{\varphi}_c + \Delta\dot{\varphi}_L = 0$ and to produce the control signal $\Delta\dot{\varphi}_c = -\Delta\dot{\varphi}_L$ that acts on the AOM. The frequency shift introduced by the AOM eliminates phase fluctuations introduced by the injection locking process. The amplified output signal is the stable optical frequency signal $\omega_0 + \omega_{AOM}$, as shown in the black frame (e).

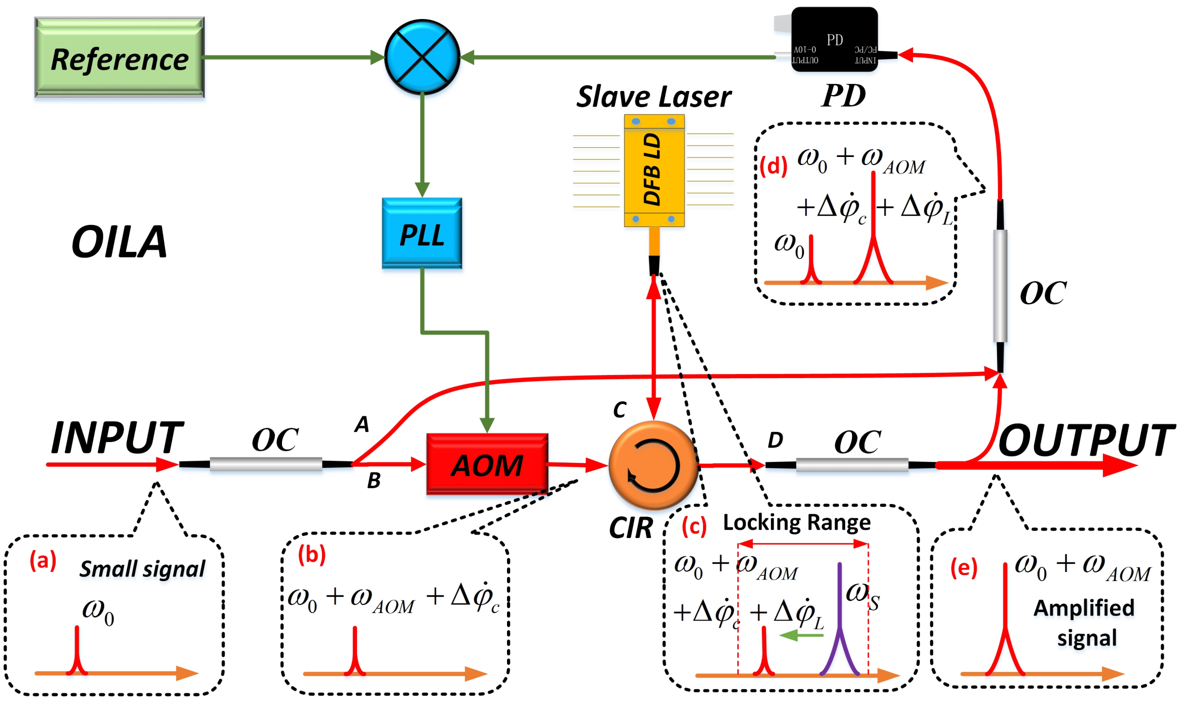

Figure 1. Optical injection locking amplifier (OILA). PD: photodiode; OC: optical coupler; CIR: circulator; AOM: acousto-optic modulator; PLL: phase locking loop.

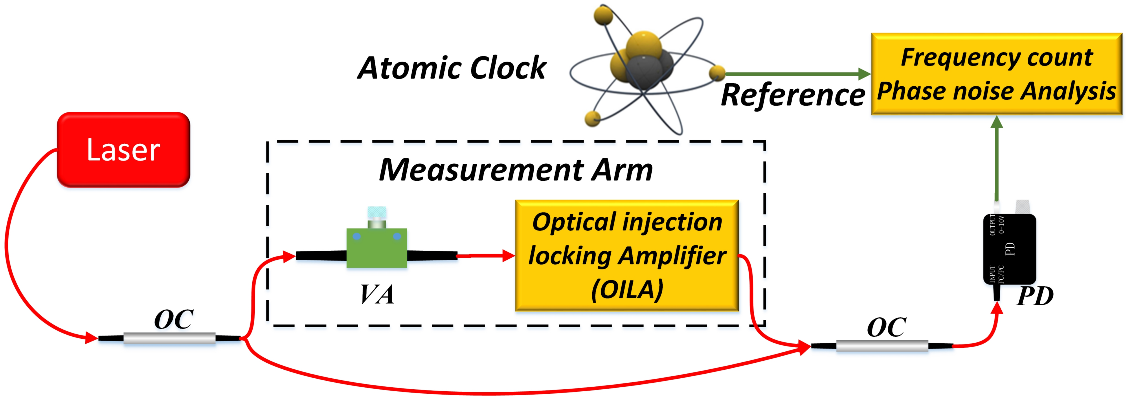

Figure 2. Set-up to measure the phase noise power spectral density (PSD) and frequency stability. VA: variable attenuator; PD: photodetector; laser: NKT-X15 narrow-linewidth fiber laser; OC: optical coupler.

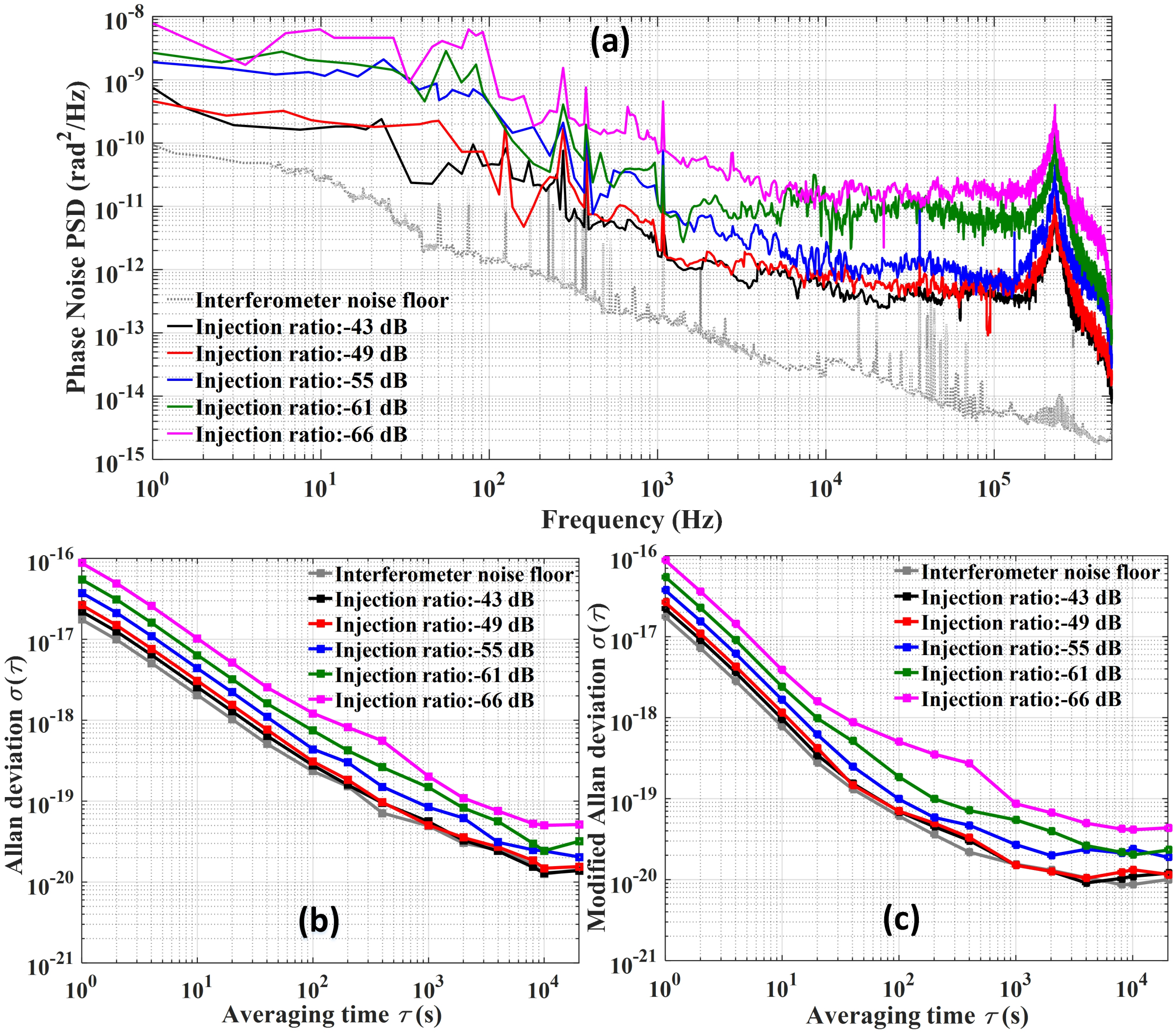

Figure 3. (a) Phase noise PSD of the optical injection locking amplifier (OILA) with different injection ratios and interferometer noise floor. (b) Allan deviation of the OILA for different injection ratios. (c) Modified Allan deviation of the OILA for different injection ratios.

We constructed an optical Mach–Zehnder interferometer to test the OILA, as shown in Figure 2. The input signal is a commercial narrow-linewidth fiber laser (NKT-X15 with 100 Hz Lorentz linewidth). The light is divided into two arms: one for the amplifier, which under different injection ratios can be controlled by tuning the variable attenuator in the measurement arm, and the light in the other functions as the reference. All PM structures in the OILA and the polarization-insensitive property of the AOM (polarization dependence losses $<$0.5 dB) help achieve long-term robustness without any polarization controller in the interferometer. The OILA frequency shift is approximately 80 MHz. Thus, the frequency of the reference arm would not be shifted. The beat signal of the reference and OILA is detected with a PD. It is then sent to a phase noise test set or a digital frequency counter to measure the phase noise or long-term stability.

The phase noise power spectral density (PSD) of the OILA for different amplification gains are shown in Figure 3(a). The interferometer noise floor is measured by removing all components from the OILA except the AOM. The noise bump in the PSDs, above 200 kHz, is the bandwidth of the optical phase-lock loop used to eliminate phase fluctuations $\Delta\varphi_L$ introduced by the optical injection locking process. The integrated phase noise of the OILA at -43 dB and -49 dB injection ratios in the frequency range from 1 Hz to 500 kHz is equal to 0.0247 rad and 0.0269 rad, respectively. Since the difference is only 0.0022 rad, we can conclude that phase fluctuations due to the optical injection locking process can still be completely suppressed when the injection ratio is reduced to nearly -50 dB. The OILA phase noise increases as the injection ratio decreases. When the OILA injection ratio reaches -55 dB, -61 dB, and -66 dB, the integrated phase noise deteriorates to 0.0461 rad, 0.0656 rad, and 0.1095 rad, respectively. The optical injection locking bandwidth is also proportional to the injection ratio. In our experiment, we measured the bandwidth to be 60 MHz when the injection ratio was -66 dB.

The integrated OILA phase noise for an injection ratio ranging from -43 dB to -66 dB corresponds to an Allan deviation (ADEV) and modified Allan deviation (MDEV) at 1 s; this is basically in accordance with the π-type dead time free frequency counter measurements, as shown in Figures 3(b) and 3(c). The MDEV follows the rule of $\tau^{-3/2}$ for an averaging time less than 20 s, indicating white phase noise at this time scale. The OILA stability degrades as the injection ratio decreases. However, the MDEV of the OILA still reaches 8.7×10^{-17} at 1 s and improves to more than 8.6×10^{-20} at 1000 s for an injection ratio of -66 dB. The results ensure that this technique can be used for achieving accurate optical frequency transfer.

OILA in optical frequency transfer

To further test the performance of the OILA, we transferred a precise optical frequency over a spool fiber link based on Doppler phase noise cancellation technology, as shown in Figure 4. The OILA under test is placed at the remote end, and the optical frequency transfer is conducted for 100 km, 120 km, 140 km, 160 km, and 180 km fiber lengths. The power attenuation

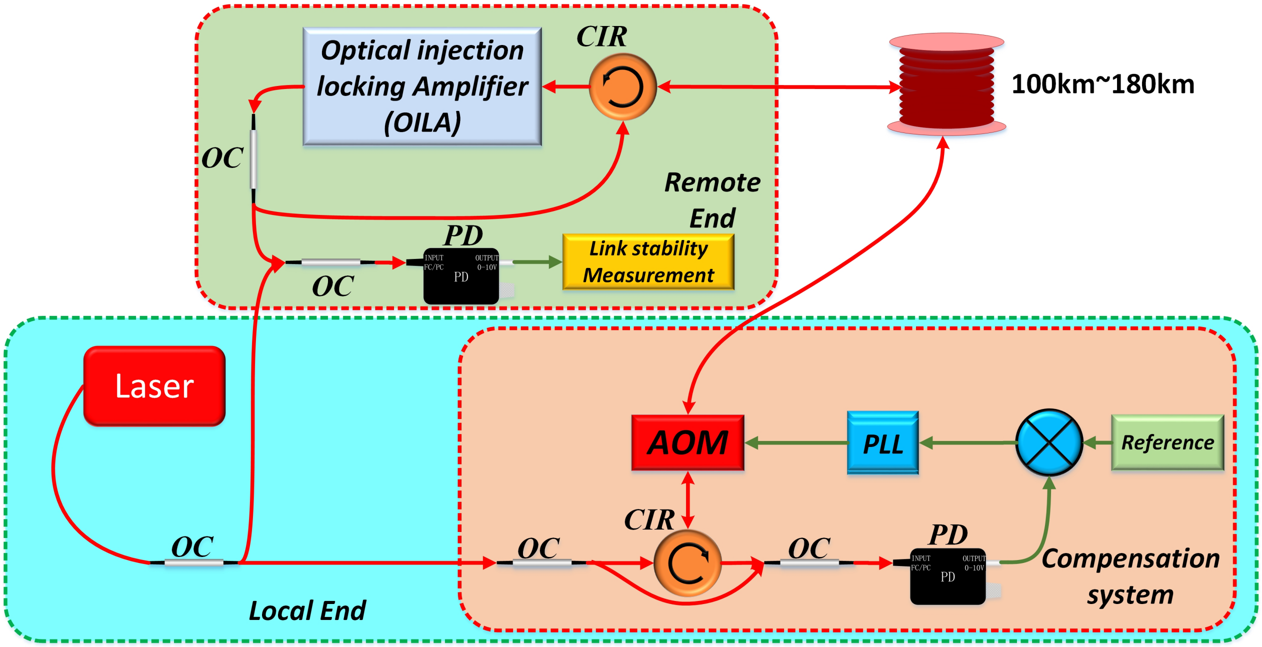

Figure 4. Schematic diagram of optical frequency transfer with fiber links ranging from 100 km to 180 km with the optical injection locking amplifier (OILA). laser: NKT-X15 narrow-linewidth fiber laser; PD: photodiode; VCO: voltage controlled oscillator; AOM: acousto-optic modulator; OC: optical coupler.

caused by these lengths causes the OILA injection ratio to correspond exactly to -43 dB, -49 dB, -55 dB, -61 dB, and -66 dB,

respectively. The transmitted signal is derived from a commercial NKT-X15 narrow-linewidth fiber laser, as mentioned earlier, with an optical frequency close to 194 THz and an output power of 6 dBm. After passing through a 30/70 optical coupler, a circulator, and an AOM, the power injected into the fiber is 0 dBm. At the remote end, the optical power attenuated due to the fiber link (we take 180 km transmission as an example) is approximately 49 dB. Inside the OILA, the attenuation of a 50/50 optical coupler, an AOM, and a circulator was 7 dB in total, implying an injection power of -56 dBm (equivalent to 2.5 nW). Since the output power from the OILA is 10 dBm, the net amplification gain in the amplifier is 59 dB (the output power of 10 dBm minus the input power of -49 dBm)[15]. Moreover, since the OILA includes an AOM for phase-locking, it can also function as a frequency shifter. Therefore, the remote end does not need to have an additional AOM for frequency-shifting. The uncompensated fiber is minimized, and all fiber-optic components are packaged in a 10 cm $\times$ 8 cm box. The box is then temperature-protected to reduce air currents that cause temperature drifts.

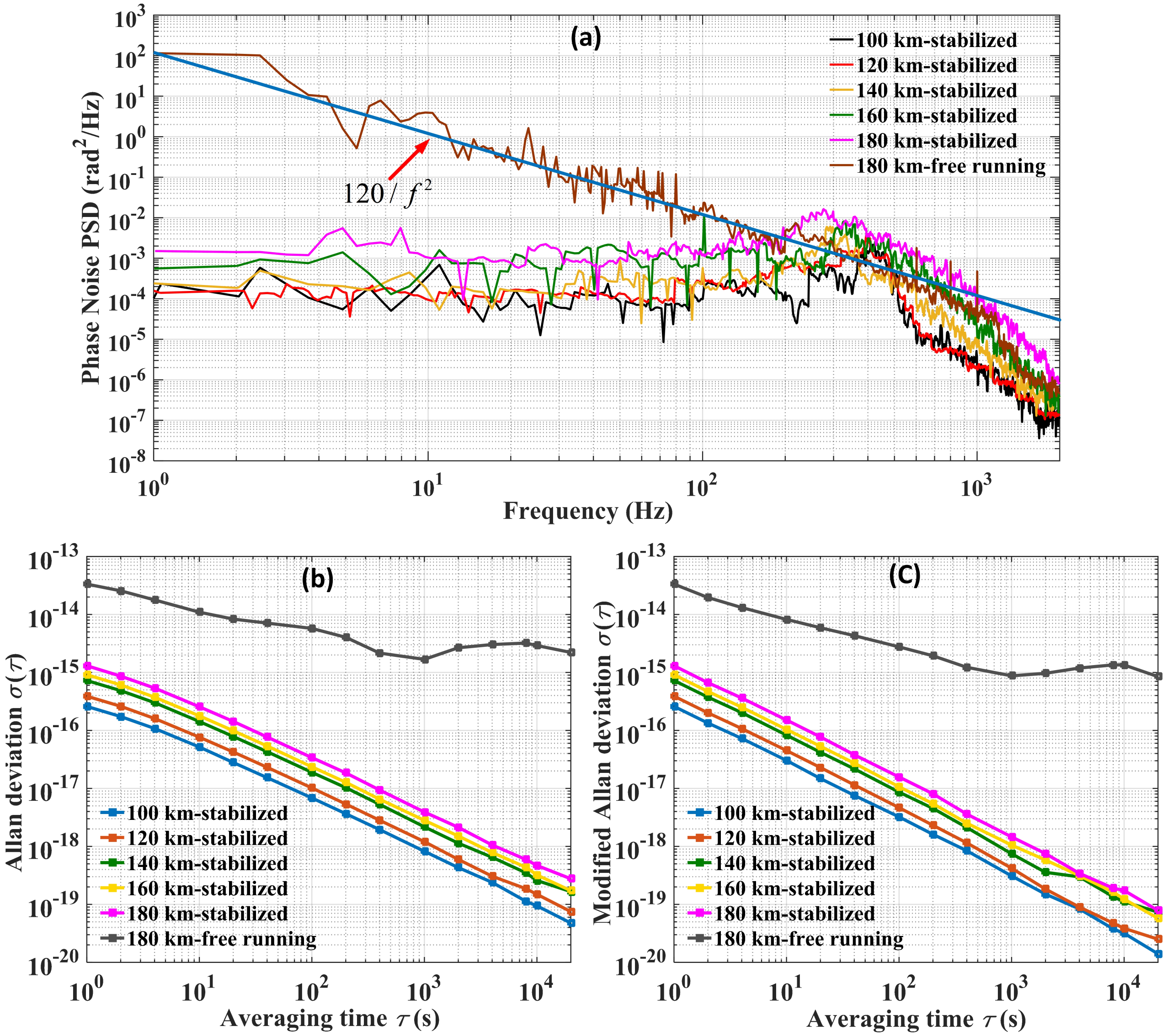

Figure 5. (a) Phase noise PSD of light in different fiber link lengths measured with the OILA. (b) Allan deviation after light traveled through different fiber link lengths measured with the OILA. (c) Modified Allan deviation after light traveled through different fiber link lengths measured with the OILA.

We use a phase noise test set to measure the noise added to the light, as shown in Figure 5(a). The phase noise added to the light after a 180 km link when the transmission system is in the free-running state follows the power law $S_{fiber}(f) = h/f^2$ for frequencies ranging from 1 Hz to 1 kHz. This indicates that the type of noise introduced by the fiber link is white phase noise. The green dashed line shows the coefficient h = 120 in this experiment. When the fiber link is stabilized, the phase noise

added to the light for a 180 km link can be remarkably suppressed to 2×10^{-3} rad^2/Hz for frequencies ranging from 1 Hz to 1 kHz. The bump at approximately 278 Hz is the bandwidth of the compensation system for the 180 km fiber link, which fits the estimated value calculated using $1/4\tau$. A comparison of the phase noise in the 180 km link between the stabilized and free-running conditions shows that the noise suppression at 1 Hz is approximately -48.67 dB, while the theoretical limit is given by[11]:

$$S_\phi^{stab}(f)/S_\phi^{free}(f) = (4\pi^2/3)(f\tau_d)^2 \tag{1}$$

Substituting $f = 1Hz$ and $\tau_d = 0.9ms$ in Eq. 1, the theoretical noise rejection limit is found to be -49.72 dB, suggesting that our transmission system is approaching the theoretical limit.

Figure 5(b) and 5(c) shows the ADEV and MDEV of the optical transmissions for different distances. Compared with the brown curve for the free-running state, the results show that noise added by the optical fiber is suppressed. After 180 km of transfer with only one amplifier, the MDEV at the remote end reaches 1.3×10^{-15} at 1 s and 1.5×10^{-18} at 1000 s.

Meantime, the MDEV for the corresponding injection ratio of -66 dB in Figure 2(d) is 8.6×10^{-20} at 1000 s. A comparison of these two MDEVs shows that the noise added by the OILA is approximately one order of magnitude smaller than that added by the stabilized fiber link. We can thus conclude that the OILA does not significantly deteriorate the transfer performance.

Discussion

We propose an OILA for optical frequency transmission applications. The phase noise and frequency stability of the OILA with different injection ratios was tested, and the results suggested that it can provide higher gain, narrower gain bandwidth, and lower noise than the bidirectional EDFA in [16]. We only used one amplifier at the remote end in our experiment on transferring a commercial NKT-X15 narrow-linewidth fiber laser carrier in a round-trip fiber link ranging from 200 km to 360 km (corresponding optical attenuation range is 72 dB to 98 dB). The amplifier was used for the mid-stage amplifier and the frequency regenerator. In this way, the output of the OILA at the remote end could be divided into two parts. One part can transfer to the next stage with 180 km fiber length, and the other returns back to the local end to determine the fiber noise[22]. Further, we did not use an additional polarization controller inside the OILA. At the remote end, the MDEV reaches values of 1.4×10^{-20} and 7.8×10^{-20} at an averaging time of 20000 s for 100 km and 180 km distances, respectively. Hence, a smaller number of amplifiers, as well as control and maintenance efforts, are required for transmission over thousands of kilometers compared to EDFAs with normal single relay distances of approximately 80 km to 100 km[16]. In the next step, the OILA will be used on an urban fiber link to distribute a cavity-stable laser carrier because the OILA is polarization-insensitive and robustness.

Methods

The optical injection locking system used as an amplifier in an optical frequency transfer system primarily needs to consider two basic parameters. The first, commonly referred to as the detuning frequency[17,18,27], is the difference between the solitary lasing frequencies of the "master" and "slave" lasers. When light from the "master" laser is injected into the "slave" laser, and the detuning frequency is less than the bandwidth of the injection locking system, the "slave" laser can establish a stable oscillation at the same optical frequency as the "master" laser, and its free-running mode is suppressed[18]. The second parameter, called the injection ratio, is the ratio of the injected optical power from the "master" laser to the output power of the free-running "slave" laser. The injection ratio can affect phase fluctuations in the injection locking system, as shown below[18]:

$$\Delta\phi_L = \sin^{-1}\left(-\frac{\Delta\omega_{inj}}{\kappa\sqrt{1+\alpha^2}}\sqrt{\frac{P_{out}}{P_{in}}}\right) - \tan^{-1}\alpha \tag{2}$$

where $\Delta\phi_L$ is the additional phase noise of the optical injection locking system, $\Delta\omega_{inj} = \omega_{ML} - \omega_{fr}$ is the detuning frequency, κ is the coupling rate, α is the linewidth enhancement factor of the slave laser, and P_{out} and P_{in} are the powers of the free-running slave and the injected signal, respectively. Inside the amplifier, the input signal power will be much less than the output power from the "slave" laser. This will create additional phase fluctuations $\Delta\phi_L$ in the amplifier, as observed from Eq. 2. We present a design for an AOM-based phase modulation and coherent detection scheme to suppress phase fluctuations. This design helps realize high gain and low noise in the amplifier.

Data availability

The datasets generated during and analyzed during the current study are available from the corresponding author upon reasonable request.

References

1. Nicholson, T. L. *et al.* Systematic evaluation of an atomic clock at 2 x 10(-18) total uncertainty. *Nat Commun* **6**, 6896 (2015).
2. Huntemann, N., Sanner, C., Lipphardt, B., Tamm, C. & Peik, E. Single-ion atomic clock with 3x10(-18) systematic uncertainty. *Phys Rev Lett* **116**, 063001 (2016).
3. Hinkley, N. *et al.* An atomic clock with 10(-18) instability. *Sci.* **341**, 1215 (2013).
4. Al-Masoudi, A., Dörscher, S., Hãfner, S., Sterr, U. & Lisdat, C. Noise and instability of an optical lattice clock. *Phys. Rev. A* **92** (2015).
5. Chou, C. W., Hume, D. B., Koelemeij, J. C., Wineland, D. J. & Rosenband, T. Frequency comparison of two high-accuracy al+ optical clocks. *Phys Rev Lett* **104**, 070802 (2010).
6. Shelkovnikov, A., Butcher, R. J., Chardonnet, C. & Amy-Klein, A. Stability of the proton-to-electron mass ratio. *Phys Rev Lett* **100**, 150801 (2008).
7. Matveev, A. *et al.* Precision measurement of the hydrogen 1s-2s frequency via a 920-km fiber link. *Phys Rev Lett* **110**, 230801 (2013).
8. Delva, P. *et al.* Test of special relativity using a fiber network of optical clocks. *Phys Rev Lett* **118**, 221102 (2017).
9. Guéna, J. *et al.* First international comparison of fountain primary frequency standards via a long distance optical fiber link. *Metrol.* **54**, 348–354 (2017).
10. Liu, Q. *et al.* Simultaneous frequency transfer and time synchronization over a 430 km fiber backbone network using a cascaded system. *Chin. Opt. Lett.* **14**, 070602 (2016).
11. Grosche, G. *et al.* Optical frequency transfer via 146 km fiber link with 10-19 relative accuracy. *Opt. Lett.* **34**, 2270–2272 (2009).
12. Terra, O. *et al.* Phase-coherent comparison of two optical frequency standards over 146 km using a telecommunication fiber link. *Appl. Phys. B* **97**, 541–551 (2009).
13. Terra, O., Grosche, G. & Schnatz, H. Brillouin amplification in phase coherent transfer of optical frequencies over 480 km fiber. *Opt. Express* **18**, 16102–16111 (2010).
14. Raupach, S. M., Koczwara, A. & Grosche, G. Optical frequency transfer via a 660 km underground fiber link using a remote brillouin amplifier. *Opt Express* **22**, 26537–47 (2014).
15. Kim, J. *et al.* Optical injection locking-based amplification in phase-coherent transfer of optical frequencies. *Opt Lett* **40**, 4198–201 (2015).
16. Predehl, K. *et al.* A 920-kilometer optical fiber link for frequency metrology at the 19th decimal place. *Sci.* **336**, 441 (2012).
17. Wei, F. *et al.* Precision and broadband frequency swept laser source based on high-order modulation-sideband injection-locking. *Opt Express* **23**, 4970–80 (2015).
18. Lau, E. K., Liang Jie, W. & Wu, M. C. Enhanced modulation characteristics of optical injection-locked lasers: A tutorial. *IEEE J. Sel. Top. Quantum Electron.* **15**, 618–633 (2009).
19. Chou, C. W., Hume, D. B., Rosenband, T. & Wineland, D. J. Optical clocks and relativity. *Sci.* **329**, 1630 (2010).
20. Lee, W.-K. *et al.* Hybrid fiber links for accurate optical frequency comparison. *Appl. Phys. B* **123** (2017).
21. Droste, S. *et al.* Optical-frequency transfer over a single-span 1840 km fiber link. *Phys Rev Lett* **111**, 110801 (2013).
22. Chiodo, N. *et al.* Cascaded optical fiber link using the internet network for remote clocks comparison. *Opt Express* **23**, 33927–37 (2015).
23. Lopez, O. *et al.* Ultra-stable long distance optical frequency distribution using the internet fiber network. *Opt. Express* **20**, 23518–23526 (2012).
24. Lopez, O. *et al.* Cascaded multiplexed optical link on a telecommunication network for frequency dissemination. *Opt. Express* **18**, 16849–16857 (2010).
25. Wu, L. *et al.* Coherence transfer of subhertz-linewidth laser light via an optical fiber noise compensated by remote users. *Opt Lett* **41**, 4368–71 (2016).

26. Deng, X. *et al.* Coherent transfer of optical frequency over 112 km with instability at the 10-20level. *Chin. Phys. Lett.* **33**, 114202 (2016).

27. Biwei Pan, B. P. *et al.* 100 gb/s all-optical clock recovery based on a monolithic dual-mode dbr laser. *Chin. Opt. Lett.* **14**, 030604–30607 (2016).

Acknowledgements

This work is supported by the Strategic Priority Research Program of the Chinese Academy of Sciences (XDB21030800), the National Natural Science Foundation of China (61775225, 61535014), and the Scientific Innovation Fund of the Chinese Academy of Sciences(CXJJ-17S010)

Author contributions statement

Zitong Feng and Fei Yang conceived the experiments and wrote this report. Both have contributed equally to this work . Haiwen Cai conceived the experiments and managed the projects. All authors carried out the experiments and contributed to the final manuscript.

Additional information

Competing Interests: The authors declare no competing interests.
