# Supplementary figures and images for: Ultra-low noise optical injection locking amplifier with AOM-based coherent detection scheme
Source: Sci Rep. 2018 Sep 3;8:13135. doi: 10.1038/s41598-018-31381-x (PMC6120882; doi:10.1038/s41598-018-31381-x)

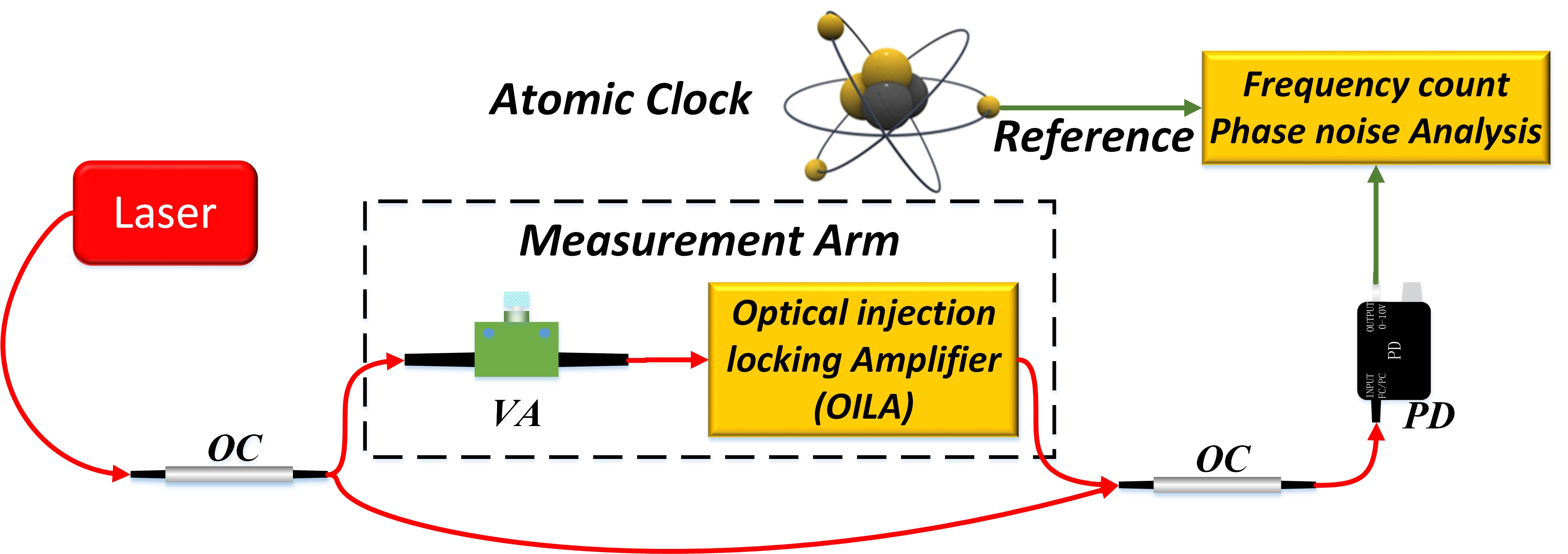

Supplement: Supplementary file 3 — LaTeX Supplementary File [file 41598_2018_31381_MOESM3_ESM.zip › SREP-18-11681/graphic/SREP-18-11681-f02.jpg]

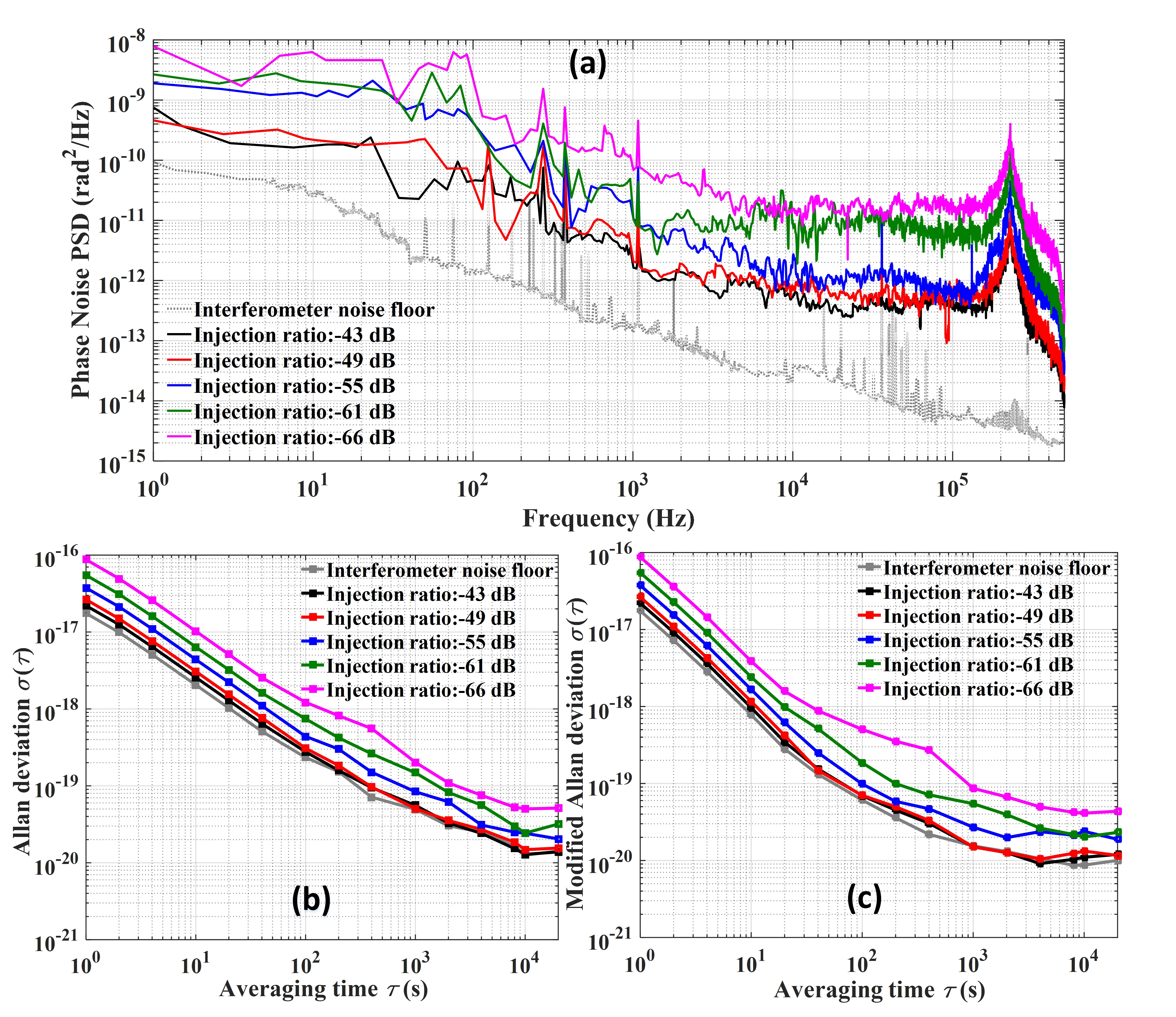

Supplement: Supplementary file 3 — LaTeX Supplementary File [file 41598_2018_31381_MOESM3_ESM.zip › SREP-18-11681/graphic/SREP-18-11681-f03.jpg]

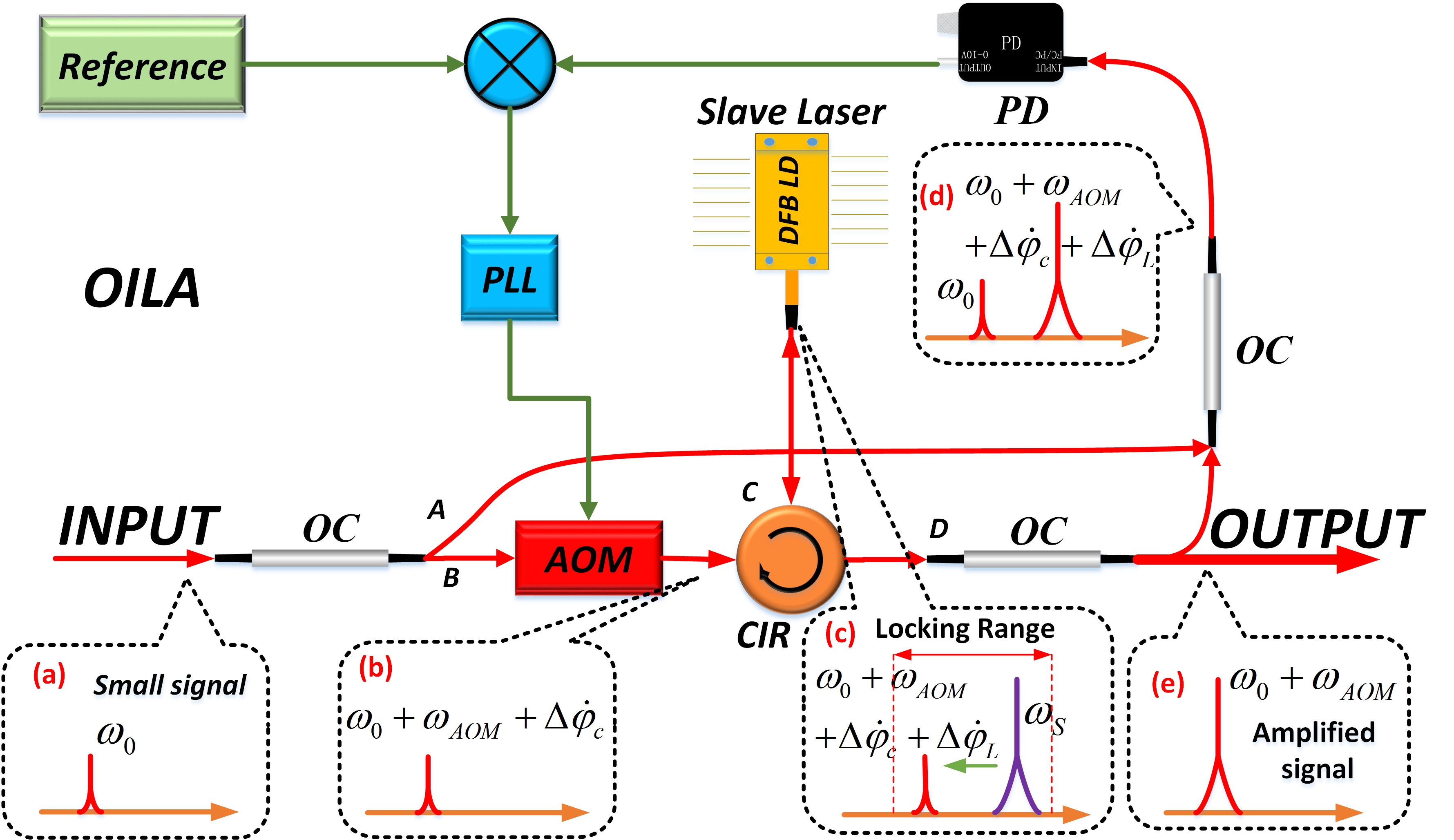

Supplement: Supplementary file 3 — LaTeX Supplementary File [file 41598_2018_31381_MOESM3_ESM.zip › SREP-18-11681/graphic/SREP-18-11681-f01.jpg]

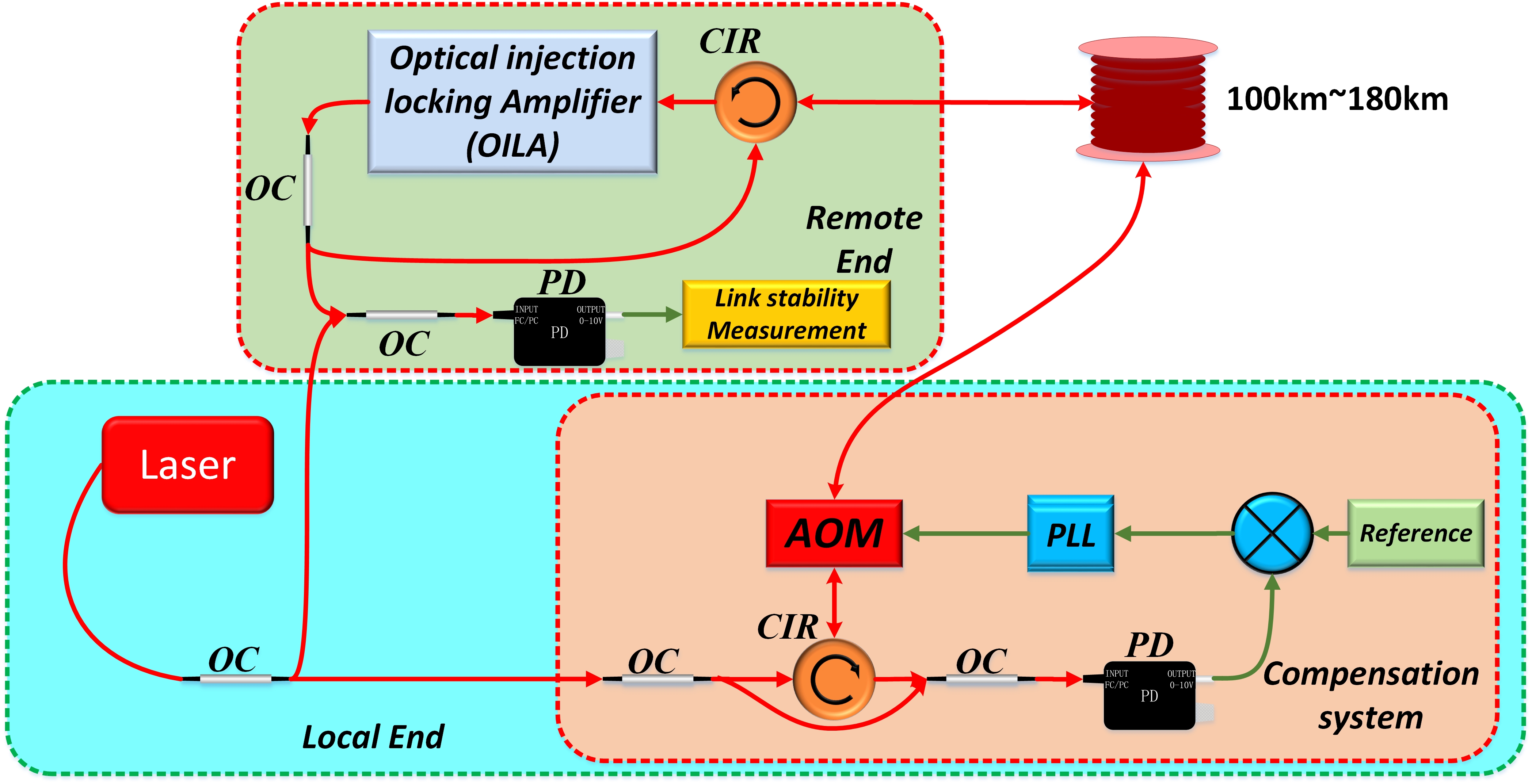

Supplement: Supplementary file 3 — LaTeX Supplementary File [file 41598_2018_31381_MOESM3_ESM.zip › SREP-18-11681/graphic/SREP-18-11681-f04.jpg]

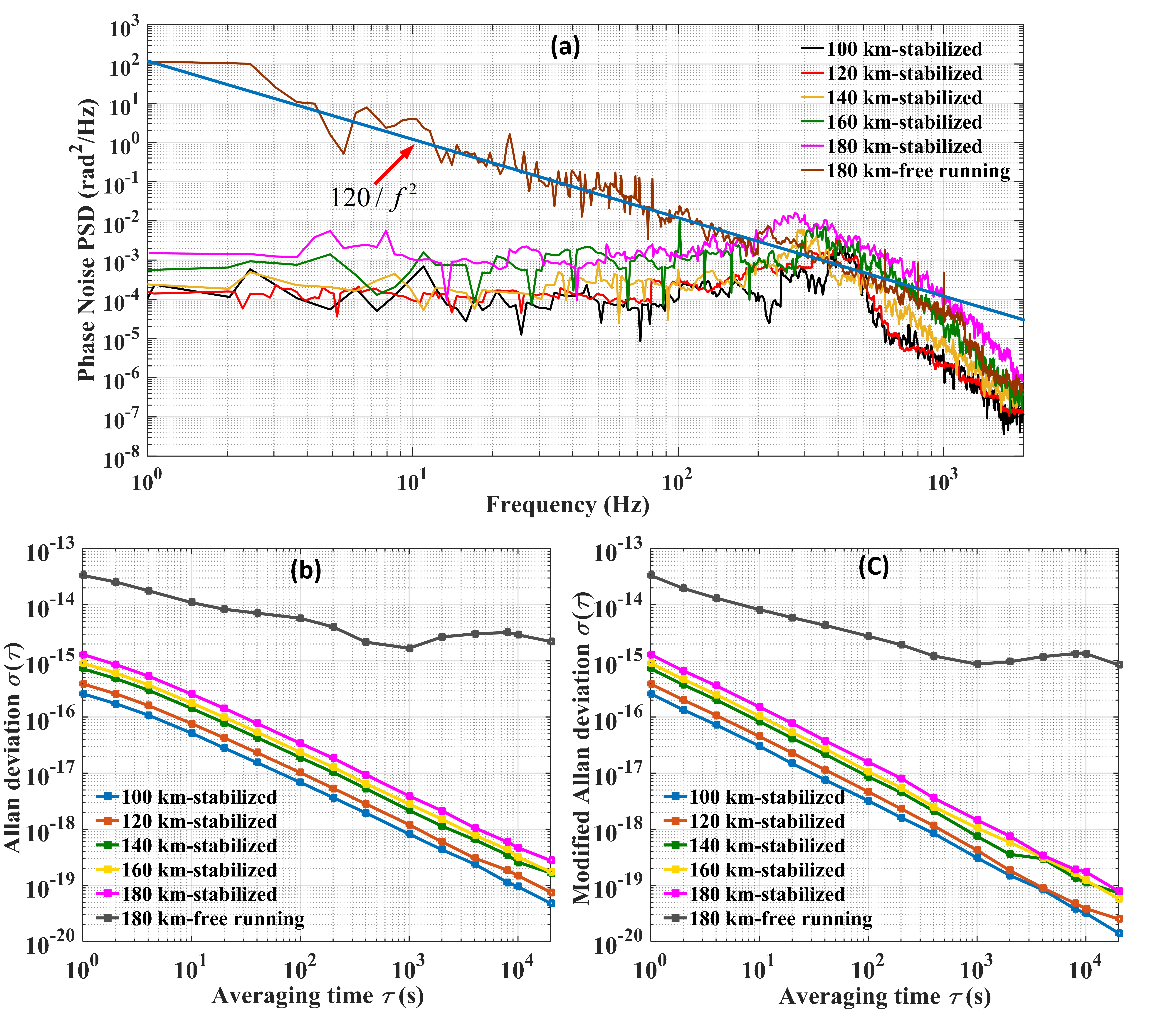

Supplement: Supplementary file 3 — LaTeX Supplementary File [file 41598_2018_31381_MOESM3_ESM.zip › SREP-18-11681/graphic/SREP-18-11681-f05.jpg]
